# Testicular Torsion in Inguinal Cryptorchidism: Report on Two Patients and Literature Review

**DOI:** 10.3390/children12111531

**Published:** 2025-11-13

**Authors:** Fabio Baldanza, Francesco Grasso, Maria Sergio, Maria Patti, Valentina Maggiore, Gregorio Serra, Mario Giuffré, Giovanni Corsello, Maria Rita Di Pace, Marco Pensabene

**Affiliations:** 1 Pediatric Surgery Unit, Department of Health Promotion, Mother and Child Care, Internal Medicine and Medical Specialties “G. D’Alessandro”, University of Palermo, 90127 Palermo, Italy; 2 Neonatal Intensive Care Unit, Department of Health Promotion, Mother and Child Care, Internal Medicine and Medical Specialties “G. D’Alessandro”, University of Palermo, 90127 Palermo, Italy

**Keywords:** undescended testis, testicular torsion, orchiectomy, orchiopexy

## Abstract

Background/Objectives: Undescended testis (UT) is a common congenital urological condition in boys, with an incidence of 2–8%. Orchiopexy is the recommended surgical treatment for UT, ideally performed before 12 months of age, with a latest window of 18 months. Testicular torsion (TT) is a pediatric urological emergency, occurring in 3.8 per 100,000 boys. While both UT and TT are common conditions individually, their co-occurrence in children under 18 months is rare and represents a challenging clinical scenario, especially when diagnosis and treatment are delayed, increasing the risk of testicular necrosis. Methods: This report describes two cases of testicular torsion in undescended testes in infants under 1 year of age. Both patients were presented to the hospital more than 24 h after symptom onset. Such delay led to the possibility of testicular salvage being lost due to necrosis. The first case involved a 10-month-old infant with bilateral undescended testis, who underwent orchiectomy after 36 h of torsion. The second case involved a 7-month-old baby, where a delayed diagnosis led to orchiectomy following 36 h of torsion. Both children were previously on the waiting list for orchiopexy, and in both cases contralateral orchiopexy was performed. A review of the literature on PubMed using the key-words “cryptorchid”, “undescended testis”, and “testicular torsion” revealed 36 cases of UT complicated by TT in children under 18 months, showing a high incidence of orchiectomies due to delayed diagnosis. Conclusions: Testicular torsion in undescended testis in children under 18 months is rare but associated with a high risk of gonadal necrosis. The key to improving testicular salvage rates is timely diagnosis and intervention, ideally within 6 h of symptom onset. Delayed presentation due to atypical clinical signs, such as inguinal swelling or nonspecific symptoms, complicates early detection and thus testicular salvage. Therefore, it is crucial for both parents and pediatricians to recognize the potential for torsion in cryptorchidic patients, emphasizing the importance of early surgical intervention, including orchiopexy, to prevent torsion and its associated complications.

## 1. Introduction

Undescended testis (UT) is the most common congenital urological condition in males, with an incidence of 2–8% [1]. According to the European Association of Urology guidelines, orchiopexy is the elective surgical treatment for UT and should be performed before 12 months of age, with an upper limit of 18 months [2]. Testicular torsion (TT) is a common pediatric urological emergency, occurring in approximately 3.8 per 100,000 boys annually [3]. However, TT in an undescended inguinal palpable testis is rare, and its occurrence in patients under 18 months is even more uncommon [4]. Here, we report on two cases of testicular torsion in undescended testes in infants under one year of age. Both patients presented to our hospital more than 24 h after symptom onset, making testicular salvage no longer feasible. Starting from this experience, we conducted a literature review to explore the clinical features, diagnostic challenges, treatment approaches, and prognosis of this condition, which carries a high risk of gonadal necrosis and its related short- and long-term effects on pediatric and adult ages health.

## 2. Materials and Methods

Cases presentation.

Two cases of testicular torsion in undescended testis were treated in our center in the last 12 months. Both patients, younger than 12 months of age, were referred by the pediatrician for a routine check-up on an undescended testicle at our institution. In both cases, orchiopexy was planned before the 12th month of age. Nevertheless, they required urgent hospitalization because of symptomatic suspected testicular torsion, occurring before the planned surgical intervention.

**Figure 1:**
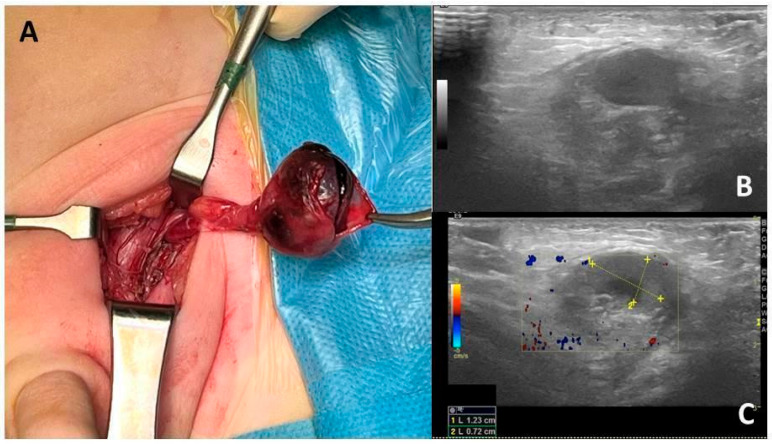
(**A**) Intraoperative appearance of necrotic left testicle. The albuginea is opened revealing necrotic parenchyma. (**B**,**C**) Ultrasound appearance of the testicle: the iso/anechoic image reveals no vascular signal and an anechoic colliquative necrosis area.

Patient 1. A 10-month-old boy, with a past medical history revealing a bilateral undescended testis, presented to a peripheral emergency Department of a small town near Palermo, Italy, with 36 h lasting inconsolable crying, left inguinal swelling and pain at palpation. A color-Doppler ultrasound was performed, revealing an immobile hypoechoic mass with no flow and an empty ipsilateral scrotal sac. The patient was transferred to our Mother and Child Department of the University Hospital of Palermo, Italy, for suspected testicular torsion of the left undescended testis. At admission, an emergency left inguinal exploration was made. A complete testicular torsion with testicle necrosis was found and a left inguinal orchiectomy was performed (Figure 1). During the same surgical intervention, a contralateral right orchiopexy was carried out. Histology confirmed the necrosis of the left testicle. Neither intra- nor post-operative complications were reported during hospitalization, leading the patient to be promptly discharged the day after surgery in good general condition. The right testicle was palpable in the scrotal sac with compensatory hypertrophy at 1 year of follow-up.

**Figure 2:**
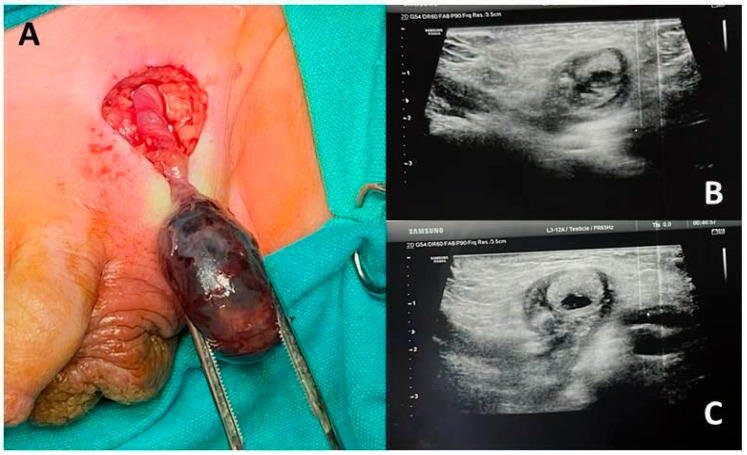
(**A**) Intraoperative appearance of left necrotic testicle and the twisted spermatic cord. (**B**,**C**) Ultrasound appearance of the testicle: the iso/anechoic image reveals an evident anechoic colliquative necrosis area.

Patient 2. A 7-month-old boy, known for having a left undescended testis since he was 5 months old, was referred to a peripheral pediatric emergency unit for 12 h of restlessness, refusal to eat, and intermittent crying. The patient was discharged with the prescription of home therapy including supplements and probiotics for a suspected abdominal colic. The mother noticed an initial improvement of symptoms. However, she then observed a left inguinal swelling and brought the child to our clinic 36 h later. A left empty scrotal sac and a hard, painful at palpation, left inguinal swelling were noted. The ultrasound confirmed the left testis within the inguinal canal, showing hypoechoic and vacuolated parenchyma and no blood flow on color-Doppler. The infant underwent left inguinal surgical exploration on general anesthesia. Orchiectomy was performed owing to a testicular necrosis secondary to complete funicular torsion, and a contralateral testicular fixation was carried out (Figure 2).

No intra- or post-operative complications were reported. The baby was discharged the day after the operation. The right testis was hypertrophic and palpable in the scrotal sac at 1 year follow-up evaluation.

**Figure 3:**
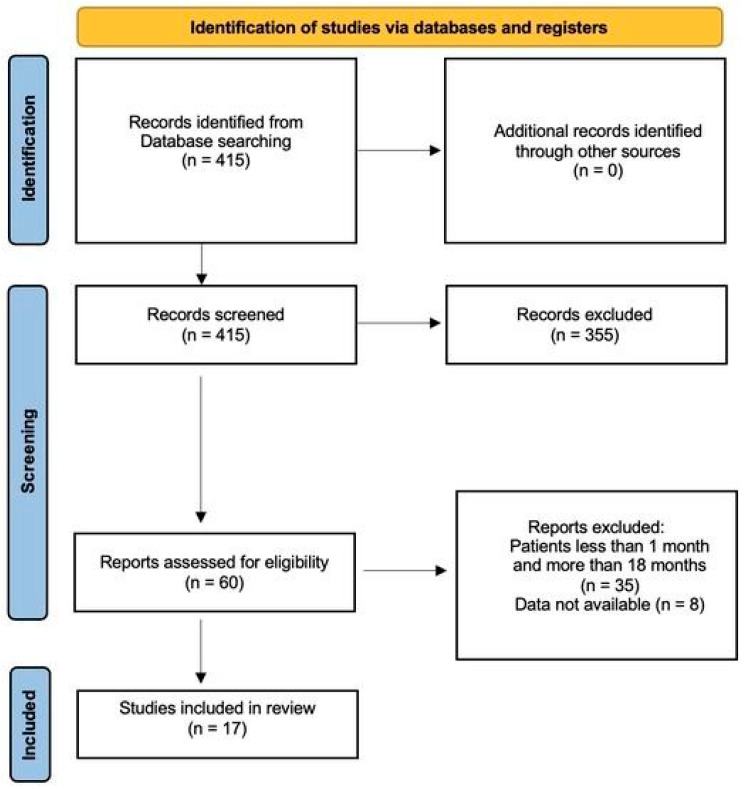
The diagram resumes the selection of papers included in the review.

Starting from the description of our patients, we performed a literature review in order to underline the clinical and instrumental characteristics of this uncommon condition. All clinical data were recorded and compared to clinical data from a literature review on similar cases. Two independent reviewers searched the PubMed database using the terms “cryptorchid”, “undescended testis”, and “testicular torsion”, and identified 415 studies based on these search criteria. The Preferred Reporting Items for Systematic Reviews and Meta-Analyses (PRISMA) criteria for systematic reviews was used to select the articles, based on specific inclusion and exclusion criteria for the eligibility [5] (Figure 3).

The literature review was completed on July 2025. The filters used were the following: Age: children 1–18 months; Language: English; Species: humans. We included all papers reporting about patients between 1 and 18 months of age with undescended testis complicated by testicular torsion, not associated with other clinical conditions. All the articles describing cases with incomplete data regarding the side involved, the clinical symptoms, the time between the onset of the symptoms and the diagnosis and the surgical treatment were excluded from the review. Papers reporting TT in UT associated with other risk factors or diseases (e.g., tumors) were not included, as well as those published in languages other than English. Articles with no available abstract were also excluded.

## 3. Results

From a literature review, 415 articles were identified on the initial search.

Of them, 17 articles were found eligible for this systematic review and provided 36 patients between 1 and 18 months of age who suffered testicular torsion in undescended testis. A total of 36 patients with unilateral cryptorchid testicular torsion were found in the literature, according to our inclusion and exclusion criteria. In 11 patients, the affected side was the right (31%), and in 25 it was the left (69%). The mean age of presentation was 9 months (1–18). The mean average time between the onset of the symptoms and the diagnosis was 24 h. The most common reported symptom was inguinal swelling on the affected side. All cases underwent a surgical inguinal canal exploration, and in 10 (28%) patients a surgical fixation of the testis was performed, while in 26 (72%) an orchiectomy was necessary, due to the necrosis of the testicle. Complete clinical manifestations and other data collected are summarized in Table 1.

## 4. Discussion

Cryptorchidism, or undescended testis, is a condition in which a testis is not palpable within the scrotum. It may be detectable during the clinical examination of the inguinal canal (palpable undescended testis) or non-palpable at all (non-palpable testis). This condition affects 1–4.6% of full-term newborn males and is associated with well-recognized maternal and perinatal risk factors, such as low birth weight, prematurity, and being small for gestational age. Despite the fact that a spontaneous migration of the testis within the scrotum frequently occurs in the first months of life, around 1% of the affected full-term babies still have an undescended testis at 1 year of age. The European Association of Urology guidelines on pediatric urology strongly recommend surgical treatment before the age of twelve months, and by eighteen months at the latest [2]. Complications of untreated cryptorchidism include inguinal hernia, low fertility, testicular cancer, and testicular torsion [22]. Moreover, testicular torsion is a well-known urological emergency that needs to be diagnosed and treated as soon as possible, since the salvage of the testis is a time-dependent outcome. TT is linked with an acute vascular event in which the spermatic cord becomes twisted on its axis, resulting in an impairment of the blood flow to and from the testicle [23]. An undescended testis appears to be at 10-times higher risk for torsion compared to the normal migrated testis [24]. According to the review, there are limited data on undescended testis related to testicular torsion for patients younger than 18 months of life. Testicular torsion is a well-defined clinical condition easily diagnosed at any emergency department. However, a twisted cryptorchid testis is not located in the scrotum and the clinical signs may be less obvious. This is the reason why physicians and parents should be informed about this possible emergency condition [4]. One major problem is the initially uncommon clinical presentation that may lead to delay in specialist consultation. Even if, from the literature review, the most common sign reported was an inguinal swelling, many authors notice further atypical and rare presentations; moreover, the inguinal swelling is not always documented at the beginning, when other nonspecific symptoms are predominant, making the spectrum of clinical manifestation more challenging and variegated [12,13,14,15,16,17,18,19,20,21]. In a case reported by Knight et al., the predominant symptom was the refusal of the patient to bear weight on his left leg from the moment he woke up in the morning [18]. Nonspecific manifestations such as pain, vomiting, and incessant crying may be misinterpreted as abdominal colic, as also occurred in one of the reported patients [12,16,17].

As observed in our review, the average time from symptom onset to diagnosis exceeded 24 h in all included cases. This duration is longer if compared to TT occurring in orthotopic testicles. Notably, studies have shown that delaying detorsion or surgical intervention beyond 4 h for torsion greater than 360°, and beyond 12 h for torsion less than 360°, is associated with an increased risk of testicular atrophy [4]. Furthermore, a recent study highlighted that, in all cases where testicular torsion was treated after 24 h, the testicle was necrotic, necessitating orchiectomy [25]. Dupond-Athénor et al. evidenced that the probability of saving the testicle is higher if the diagnosis is made within 6 h from symptoms onset [1].

In the series of Singal et al., the time at diagnosis from the onset of symptoms was over 36 h in all cases [20]. These results are consistent with both the findings of the literature review and our experience; actually, in our series, the nonspecific clinical presentation of both patients led to a delay in the initial clinical evaluation and, consequently, in surgical treatment, and eventually to gonadal loss. Such lateness in surgical treatment might be due to the low awareness of parents about this rare complication of an undescended testis [10]. Diagnostic process can be supported by ultrasound examination with power- and color-Doppler integration: the affected testis is edematous, and may present heterogeneous parenchyma and lack blood flow at Doppler; as observed in our patients, in cases of testicular torsion > 24 h, ultrasound images may show hypo/hyperechoic areas and even frankly anechoic ones. These findings have a negative prognostic significance as they represent an indirect sign of liquefactive necrosis [15]. Although ultrasound is the first-line imaging modality for the evaluation of testicular torsion, it has decreased accuracy in the evaluation of torsion in undescended testis [26]. Some Authors reported other imaging tools being used, such as computed tomography (CT) scan, magnetic resonance imaging (MRI), and 99mTc-Scyntigraphy, to reach diagnosis [16,17].

Others highlighted that proper medical history collection and accurate clinical examination may be enough to suppose a diagnosis, and not to postpone the surgical treatment. [10,11,12,13,14,15,16,17,18,19,20,21]. According to the literature review, TT in UD shows a poor prognosis for the testicle, since most of the described cases, as well as the presented ones, presented a low salvage rate of the testicle, due to the delay in the diagnosis and in surgical treatment. A total of 25/26 patients undergoing orchiectomy were treated over 6 h from the onset of the symptoms, which has been shown to be the optimal time window to increase salvage rate [14,15]. On the other hand, 4/10 patients undergoing orchiopexy were treated over 6 h from onset, confirming that saving a twisted testicle is a time-dependent option.

Orchiopexy is the surgical treatment for the undescended testicle, and prevents the risk of complications. As reported in EAU guidelines, orchiopexy should be performed within the first 12 months of age [2]. Nonetheless, in many centers the mean age of patients for elective orchiopexy is more than 2 years [27].

Even if the association between the UT and TT is uncommon, the risk of loss of the testis seems to be higher if TT occurs in a UT [4,12,15,20]. From our review, the median age of presentation was 9 months, but, of the 36 patients included, 83% were under 1 year of age, suggesting early orchiopexy to prevent orchiectomy secondary to TT.

## 5. Conclusions

In conclusion, TT can also occur in UT and, although rare, this condition carries delay in a proper diagnosis, and thus a poor prognosis for the testicle. Clinical suspicion should arise in patients with UT and nonspecific symptoms, and torsion should always be ruled out, since every effort should be made to save the testicle in these cases. The consequences of orchiectomy can manifest even years later, as the appearance of the external genitalia plays a significant role in shaping a satisfactory self-image, as occurs for other congenital defects, during childhood and adolescence [28,29,30,31,32]. In order to ensure the patient the best treatment options, a complete medical history and physical examination of the genitalia should be performed, to lead to a diagnosis even in the early stage of the TT, when only nonspecific symptoms such as crying, vomiting, and pain occur.

Parents and pediatricians should be aware about this possible complication to guarantee a rapid diagnosis and treatment. Orchiopexy is the elective surgical treatment for undescended testis, and it should be performed in a timely manner to reduce the risk of loss of the testis.

## 6. Future Prospects

The optimal timing of orchiopexy remains a subject of clinical importance in the management of undescended testes (UDT). Current evidence strongly supports early surgical intervention, ideally between 6 and 12 months of age, to optimize fertility potential and reduce the risk of testicular malignancy. Early orchiopexy has been shown to promote normal germ cell development, preserve Sertoli and Leydig cell function, and facilitate reliable testicular examination during follow-up.

In contrast, waiting for spontaneous testicular descent beyond six months of age is generally discouraged. Although spontaneous descent may occur in neonates—particularly in premature infants—most cases that have not descended by this time are unlikely to do so subsequently [2,27,33].

Hence, it would be advisable for pediatricians to refer patients with UT to a pediatric surgeon within the first months of life, allowing the specialist to determine the most appropriate clinical and therapeutic management for each patient.

This paper highlights a rare but serious complication that may be underrecognized, even among specialists. Timely orchiopexy and comprehensive education of both parents and pediatricians are essential to reduce the risk of testicular loss.

## Figures and Tables

**Table 1:** The table resumes and compares main characteristics of reviewed cases.

Author	Age (Months)	Side	Onset	Symptoms	Treatment
Zvizdic et al. [4]	2	L	4 h	Restlessness, decreased intake, and inguinal swelling	Orchiopexy
	5	L	60 h	Restlessness, inguinal swelling, and pain	Orchiectomy
Andrade et al. [6]	6	R	24 h	Vomiting and inconsolable crying	Orchiectomy
Candocia et al. [7]	7	L	5 h	Inguinal swelling, inconsolable crying	Orchiectomy
Deng et al. [8]	4	L	24 h	Abdominal pain, inguinal swelling, and inconsolable crying	Orchiectomy
	10	L	18 h	Pain, vomiting, and inguinal swelling	Orchiectomy
Carvalho et al. [9]	5	L	24 h	Inguinal swelling	Orchiectomy
Komarowska et al. [10]	6	L	96 h	Pain, fever, and inguinal swelling	Orchiectomy
Lio K. et al. [11]	4	R	48 h	Restlessness, inguinal swelling, and fever	Orchiectomy
Pogorelic et al. [12]	12	L	36 h	Inguinal swelling, pain, and inconsolable crying	Orchiectomy
Erdogan et al. [13]	8	L	10 h	Inguinal swelling and inconsolable crying	Orchiectomy
Naouar et al. [14]	12	R	3 h	Inguinal swelling and inconsolable crying	Orchiopexy
Kargl et al. [15]	9	R	48 h	Vomiting and inguinal swelling	Orchiectomy
	11	R	8 h	Inguinal swelling and restlessness	Orchiectomy
	7	L	24 h	Inguinal swelling and pain	Orchiectomy
	9	R	12 h	Inguinal swelling and pain	Orchiopexy
	12	L	24 h	Inguinal swelling and pain	Orchiectomy
	4	L	6 h	Inguinal swelling and pain	Orchiopexy
	6	L	12 h	Inguinal swelling and pain	Orchiectomy
	1	R	16 h	Inguinal swelling	Orchiectomy
	11	L	12 h	Restlessness and inguinal swelling	Orchiectomy
	12	R	6 h	Asymptomatic	Orchiopexy
Stoykov et al. [16]	18	L	6 h	Perineal swelling	Orchiopexy
Shayegani et al. [17]	9	L	13 h	Inconsolable crying	Orchiopexy
Knight et al. [18]	14	L	12 h	Refusing to bear weight	Orchiectomy
Mowad et al. [19]	5	L	48 h	Inguinal swelling	Orchiectomy
Singal et al. [20]	4	L	48 h	Inguinal swelling and inconsolable crying	Orchiectomy
	4	L	48 h	Inguinal swelling	Orchiectomy
	5	L	36 h	Inguinal swelling	Orchiectomy
	11	R	48 h	Inguinal swelling	Orchiectomy
	10	L	36 h	Inguinal swelling	Orchiectomy
Sener et al. [21]	16	R	7 h	Inconsolable crying, inguinal swelling, and pain	Orchiopexy
	15	L	11 h	Inconsolable crying, inguinal swelling, and pain	Orchiectomy
	18	L	22 h	Inconsolable crying, inguinal swelling, and pain	Orchiectomy
	16	L	8 h	Inconsolable crying, inguinal swelling, and pain	Orchiopexy
	8	R	4 h	Inconsolable crying, inguinal swelling, and pain	Orchiopexy
Baldanza et al.	10	L	36 h	Inconsolable crying, inguinal swelling, and pain	Orchiectomy
	7	L	36 h	Inconsolable crying, inguinal swelling, and pain	Orchiectomy

## Data Availability

Not applicable.

## Abbreviations

The following abbreviations are used in this manuscript:

UT	Undescended testis
TT	Testicular torsion
CT	Computerized tomography
MRI	Magnetic resonance imaging
